# Role and potentialities of bacteria associated with *Tuber magnatum*: A mini-review

**DOI:** 10.3389/fmicb.2022.1017089

**Published:** 2022-10-06

**Authors:** Pamela Monaco, Gino Naclerio, Antonietta Mello, Antonio Bucci

**Affiliations:** ^1^Department of Biosciences and Territory, University of Molise, Pesche, Italy; ^2^Institute for Sustainable Plant Protection (IPSP), Turin Unit, National Research Council, Turin, Italy

**Keywords:** ectomycorrhizal fungi, truffle, *Tuber magnatum*, microbial communities, bacteria

## Abstract

Among the hypogeous ectomycorrhizal fungi, the white truffle *Tuber magnatum* Picco is the species of greatest interest, both from an ecological and economic point of view. The increasing market demand of the precious white truffle along with the fall in its natural production led to a growing interest in cultivation techniques and encouraged truffle growers and researchers to deeper investigate factors that could affect and improve *T. magnatum* productivity. In this context, microbial communities play a central role. Indeed, in the last few years, the hypothesis of a potential link between microbial community composition and truffle orchard productivity is arousing a greater attention. Moreover, since the value of the prized *T. magnatum* can vary in relation to its provenience, the need to define a reliable tracking system is also emerging and bacteria appear to be a promising tool. Accordingly, the present mini-review summarises the knowledge currently available on *T. magnatum* microbial communities, focusing on the role of truffle-associated bacteria and highlighting similarities and differences between samples of different origin, to address the following issues: (i) Is there a correlation between microbial taxa and truffle ground productivity? (ii) Can bacteria actually be used as markers of *T. magnatum* geographic origin? The identification of microorganisms able to promote *T. magnatum* formation may represent an important advance in the field of truffle farming. Similarly, the detection of bacterial taxa that can be used as markers of *T. magnatum* origin could have a considerable impact on truffle industry and trade, even at local scale.

## Introduction

Most of terrestrial plant roots are colonised by mycorrhizal fungi; indeed, it is estimated that more than 90% of all plant species and over 6,000 fungal species in the *Glomeromycotina*, *Ascomycotina*, and *Basidiomycotina* subdivisions are involved in the formation of mycorrhizas, symbiotic associations between fungi and plant roots (Bonfante and Anca, 2009; Bonfante and Genre, 2010). Mycorrhizal fungi play a key role in plant ecosystems since they improve the nutrient status of the host plant, providing minerals and increasing water absorption from the soil, and confer resistance to stress and disease (Bonfante and Genre, 2010; Mello and Balestrini, 2018; Lanfranco and Bonfante, 2022). Moreover, they allow an efficient horizontal transfer of nutrients through the development of the so-called wood-wide web, an extensive hyphal network in the soil that connects different plants (Bonfante and Genre, 2010). At the same time, for its growth and reproduction the fungus needs the host plant, which provides carbon compounds (Mello and Balestrini, 2018). Based on the taxonomic position of plant and fungal partners and anatomical traits, mycorrhizas are commonly divided into two main categories: ectomycorrhizas and endomycorrhizas, depending on whether the fungus colonises the intercellular spaces or develops inside root cells.

A well-known example of ectomycorrhizal fungi is given by truffles (*Tuber* spp.), hypogeous fungi whose fruit bodies sequester the spores and develop underground, bringing benefits to the forest ecosystems and to the host plants (Trappe and Claridge, 2010; Monaco et al., 2020a). In addition to their ecological role, some truffle species (as for instance *Tuber magnatum* Picco, *T. melanosporum* Vittad., *T. borchii* Vittad., and *T. aestivum* Vittad.) are of considerable economic and commercial importance. Moreover, truffle hunting and collection practise has recently been recognised as Intangible Cultural Heritage of Humanity^1^ due to its naturalistic, cultural, and anthropological relevance.

Among the more than 180 *Tuber* species currently known, *T. magnatum* Picco, the so-called Italian white truffle, is the species of greatest interest and with the highest economic value (Bonito et al., 2010, 2013; Vita et al., 2015; Benucci and Bonito, 2016). Indeed, in the final stage of its complex life cycle, this ectomycorrhizal ascomycete of the *Pezizales* order produces edible fruiting bodies that can be considered one of the most expensive foods in the world, reaching a cost of thousands of euros per kilogram (Riccioni et al., 2016; Patel et al., 2017; Daba et al., 2019; Laruccia et al., 2020; Monaco et al., 2021a). Multiple factors contribute to the exorbitant prices of *T. magnatum* ascomata. On one side, their valuable culinary properties and unique organoleptic qualities ensure that market demand and truffle trade are constantly expanding. On the other side, the annual production of *T. magnatum* results generally insufficient to satisfy these needs (Bach et al., 2021), because of the restricted distribution range, limited seasonal availability, and difficulties in cultivation (Marjanović et al., 2015; Belfiori et al., 2020; Zambonelli et al., 2021). The indiscriminate collection, climate change, deforestation, and wildfires are added to this (Perlińska-Lenart et al., 2020; Marozzi et al., 2022), putting *T. magnatum* at risk of extinction. In this context, the full understanding of the factors that influence the life cycle of the precious white truffle and promote the development of fruiting bodies is of fundamental importance, also because this species is considered an indicator of a healthy environment. Among these factors, bacterial communities play a central role (Amicucci et al., 2018; Monaco et al., 2020a; Sillo et al., 2022). Accordingly, in the present mini-review, we summarised the knowledge currently available on *T. magnatum* associated bacterial communities, highlighting similarities and differences between samples of different origin, to address the following issues: (i) Is there a correlation between microbial taxa and truffle ground productivity?/Are there microbial taxa related to a higher truffle production? (ii) Can bacteria actually be used as markers of *T. magnatum* geographic origin?

## Role of bacteria associated with truffles (*Tuber* spp.)

Interactions between fungi and bacteria have long been studied in mycology (Waksman, 1927). Some bacterial species are beneficial to fungi, promoting the establishment of mycorrhizas and fruiting body development (Aspray et al., 2006; Frey-Klett et al., 2007, 2011), others can be responsible for fungal pathogenicity, may control sporulation (Partida-Martinez and Hertweck, 2005; Benucci and Bonito, 2016) or have a detrimental effect on mycelium development (Barbieri et al., 2005).

These interactions also concern *Tuber* species, which are in close contact with microorganisms throughout their life cycle. Indeed, truffles harbour and interact with complex microbial communities of bacteria, yeasts, and filamentous fungi, as well as viruses (Stielow and Menzel, 2010; Splivallo et al., 2015, 2019; Vahdatzadeh et al., 2015, 2019; Benucci and Bonito, 2016; Ratti et al., 2016). A combination of culture-dependent and independent methods and different techniques (from denaturing gradient gel electrophoresis to high-throughput sequencing) has been employed to investigate the truffle microbiota (Mello et al., 2013; Vahdatzadeh et al., 2015). Considerable differences emerged depending on the method of analysis used (Bonfante and Anca, 2009); whilst molecular techniques allow to describe more accurately the bacterial communities as a whole, culture-dependent methods have some limitations since microorganisms that can be cultivated in the laboratory represent only a small fraction of the total diversity existing in nature (Stewart, 2012; Perlińska-Lenart et al., 2020).

Bacteria can heavily colonise truffle ascocarps, both the inner tissues (gleba) and the surface (peridium), reaching a density from millions to billions of cells per gram (dry weight; Reale et al., 2009; Splivallo et al., 2015, 2019; Vahdatzadeh et al., 2015, 2019). Peridium and gleba seem to attract specific bacterial genera (Barbieri et al., 2016), which appear to be selected from the soil communities during the early stage of truffle formation. In fact, it is believed that soil bacteria colonise *Tuber* primordia before the differentiation of ascocarpic tissues occurs, when the primordium is directly in contact with soil. Subsequently, after the differentiation of the peridium, bacteria would be trapped in the gleba, partly protected from soil exchanges by the peridium that, on the contrary, remains in contact with ground throughout the ascocarp development (Antony-Babu et al., 2014; Monaco et al., 2020a; Vita et al., 2020).

Multiple factors can influence the composition of the truffle-associated microbial communities. Several studies highlighted variations in the microbiota structure related to the different *Tuber* species, life cycle stage of the fungus (e.g., mycorrhizas vs. fruiting bodies), ascocarp maturation, tissue specificity (gleba vs. peridium), storage period, collection site, harvesting season, and environmental conditions (Vahdatzadeh et al., 2015; Monaco et al., 2021b; Niimi et al., 2021a; Sillo et al., 2022). Therefore, *Tuber* species provide diverse microhabitats hosting complex and changeable microbial communities, involved in numerous functions. In particular, bacteria—the third partner of the symbiosis between *Tuber* and its host plant—seem to play a central role in the complex biological processes of signalling and nutrient exchanges involving hyphae, ectomycorrhizas, and ascocarps (Barbieri et al., 2016). In exchange for water and nutrients, they produce biostimulants (e.g., phytohormones and specific amino acids), promote the growth of mycelium and ectomycorrhiza formation (Sbrana et al., 2002; Frey-Klett et al., 2007), and participate in the development and maturation of truffle fruiting bodies (Mello et al., 2010; Antony-Babu et al., 2014; Amicucci et al., 2018). Bacteria may improve fungal nutrition (of both primordium and developed ascocarp) by enhancing the availability of some elements (N, P, micronutrients) through their nitrogen fixing activity, chemical transformation, phosphate solubilisation, and chelating compound production (Pavić et al., 2011, 2013). Moreover, some bacterial taxa could be involved in spore germination and in the opening of asci and ascospore release thanks to their cellulolytic and chitinolytic activities (Gazzanelli et al., 1999; Pavić et al., 2011); others inhibit/counteract the growth of pathogens and contaminating fungi by producing antimicrobial substances. Microorganisms are also partly responsible for truffle aroma since they synthesise sulphur volatile compounds that, besides determining the organoleptic properties of fruiting bodies, attract mammals. In this way, bacteria take indirectly part in the dissemination of truffle spores, confirming their key role in the life cycle of the fungus (Splivallo et al., 2011, 2015; Splivallo and Ebeler, 2015).

## The microbial communities of *Tuber magnatum*

To date, there are about a dozen papers describing the microbial communities associated with the precious white truffle *T. magnatum* Picco. Most studies have focused on bacteria, whilst fungi have been little investigated, probably because of the difficulties related to the massive presence of *Tuber* DNA that can interfere and hinder the amplification, sequencing and detection of “exogenous fungi” from the gleba (Niimi et al., 2021a; Marozzi et al., 2022).

Bacterial communities associated with *T. magnatum* ascocarps were described for the first time by Barbieri et al. (2007), who reported important differences in the composition of the microbiota depending on the analysis method (Table 1). Indeed, whilst most of the isolated strains were fluorescent pseudomonads belonging to the *γ-Proteobacteria* class, 16S rDNA clone library sequencing and fluorescence *in situ* hybridization (FISH) showed a predominance of α-*Proteobacteria*, mainly represented by members of *Sinorhizobium*/*Ensifer* and *Rhizobium*/*Agrobacterium* groups, as well as *Bradyrhizobium* species (e.g., *B. elkanii*). Subsequent studies based on high-throughput sequencing techniques confirmed these results, highlighting that the bacterial communities associated with white truffle ascomata from diverse countries (Italy, Croatia, Serbia, and Hungary) were dominated by α-*Proteobacteria* and, in particular, by *Bradyrhizobium* species, regardless of their provenience and maturation degree (Monaco et al., 2021b; Niimi et al., 2021a,b; Marozzi et al., 2022). However, a different outcome emerged from the analysis of some *T. magnatum* collected in Tuscany (Central Italy), with gleba communities characterised by a prevalence of *γ-Proteobacteria* and *Pedobacter*, *Burkholderia*, *Pseudomonas*, and *Flavobacterium* as dominant taxa (Sillo et al., 2022).

**Table 1:** Main bacterial taxa associated with *Tuber magnatum* ascocarps reported in literature.

***Tuber magnatum* ascocarp-associated bacteria**			
**Bacterial taxa**	**Analysis method**	**Truffle geographic origin**	**References**
Fluorescent pseudomonads (*γ-Proteobacteria*)	Culture-dependent	North-central Italy	Barbieri et al., 2007
*Sinorhizobium*, *Rhizobium*, and *Bradyrhizobium* spp. (*α-Proteobacteria*)	Molecular approaches (16S rDNA clone library sequencing, FISH)		
*Sphingobium* sp. (*α-Proteobacteria*)	Culture-dependent	/	Pavić et al., 2011
*Curtobacterium flaccumfaciens*, *Rhodococcus* sp. (*Actinomycetia*)	Culture-dependent	Western Serbia	Pavić et al., 2013
*Bradyrhizobium*, *Mesorhizobium*, *Phyllobacterium*, *Allorhizobium-Neorhizobium-Pararhizobium-Rhizobium*, *Devosia*, *Ensifer*, *Sphingopyxis*, *Rhodopseudomonas* (*α-Proteobacteria*), *Flavobacterium* (*Flavobacteriia*), *Pedobacter* (*Sphingobacteriia*), *Polaromonas*, *Variovorax* (*β-Proteobacteria*), and *Chitinophaga* (*Chitinophagia*)	High-throughput sequencing	Central-Southern Italy (Molise)	Monaco et al., 2021b
*Bradyrhizobium*, *Phyllobacterium* (*α-Proteobacteria*), *Chitinophaga* (*Chitinophagia*), and *Pseudomonas* (*γ-Proteobacteria*)	High-throughput sequencing	Italy (Abruzzo); Hungary; Serbia; Croatia	Niimi et al., 2021a
*Bradyrhizobium*, *Rhizobium*, *Ensifer* (*α-Proteobacteria*), *Pseudomonas* (*γ-Proteobacteria*), *Polaromonas* (*β-Proteobacteria*), and *Chitinophaga* (*Chitinophagia*)	High-throughput sequencing	Central Italy (Umbria and Tuscany)	Marozzi et al., 2022
*Pedobacter* (*Sphingobacteriia*), *Burkholderia* (*β-Proteobacteria*), *Pseudomonas* (*γ-Proteobacteria*), *Flavobacterium* (*Flavobacteriia*) and *Phenylobacterium* (*α-Proteobacteria*; peridium)	High-throughput sequencing	Central Italy (Tuscany)	Sillo et al., 2022

In the last few years, the hypothesis of a potential relationship between microbial community composition and truffle orchard productivity is arousing increasing interest. Mello et al. (2010) first analysed the soil fungal and bacterial communities within a natural truffle ground of *T. magnatum* in Montemagno (Piedmont, Northern-Italy) in relation to its productive niches, with the aim of identifying potential “productivity markers.” They found that no specific fungal populations could be associated with productive/unproductive sites, even if *Mortierella* genus and *Fusarium oxysporum* appeared more abundant in productive soils. On the contrary, a potential link between the presence of *T. magnatum* ascocarps and the γ-Proteobacterium *Moraxella osloensis* was observed, indicating this bacterium as a promising marker of truffle productivity. A more recent study highlighted that microorganisms appear to be a better indicator of the truffle ground productive potential than chemical parameters, such as pH and phosphorus concentration. A decrease in bacterial diversity from *T. magnatum* unproductive to productive soils has been detected with prokaryotes belonging to *Nitrososphaerales* and *Gemmatales* orders almost exclusively present in unproductive lands (Sillo et al., 2022). However, in natural conditions, different soil microbial communities can sustain white truffle production as proved by the significative differences observed in the structure of the fungal and bacterial communities between *T. magnatum* productive sites (Marozzi et al., 2022). Fruiting body communities showed a lower diversity compared to the surrounding bulk soil, with a further reduction from peridium to gleba (Sillo et al., 2022). Indeed, the truffle microbial communities converge on few selected taxa (mainly represented by *Mortierella* for fungi, *Bradyrhizobium*, *Rhizobium*, *Pseudomonas*, *Ensifer*, *Polaromonas*, *Pedobacter*, *Chitinophaga*, and *Phyllobacterium* for bacteria), which form the “core microbiota” of *T. magnatum* ascocarps (Monaco et al., 2021b; Niimi et al., 2021a; Marozzi et al., 2022). Factors driving this selection have not yet been fully understood (Vahdatzadeh et al., 2015). A selective pressure on microorganisms related to specific potential functions and an evolutionary adaptation of some bacterial taxa to the genus *Tuber* could explain the reduction of diversity and the exclusive presence of certain microbial groups inside the fruiting bodies (Marozzi et al., 2022; Sillo et al., 2022). Therefore, the constant occurrence of defined bacterial genera in *T. magnatum* ascocarps with different geographical origin confirms/demonstrates their central role in truffle ecology and life cycle. On the other hand, since several biotic and abiotic factors drive the composition of microbial communities, “variable taxa” can complete the truffle microbiota contributing to determine the differences observed even at local scale (Monaco et al., 2021b). For example, the environmental conditions of the hypogeous habitat can affect (promoting or not) the bacterial colonisation of ascocarps and lead to changes in the microbiota structure, as reported by Amicucci et al. (2018) for pigment-producing bacteria that, in response to specific environmental stimuli, could release secondary metabolites (e.g., carotenoids), which seem to be responsible for the chromatic alteration (reddish patches) often observed within *T. magnatum* ascomata.

## Discussion

In the last years, the increasing market demand of the precious white truffle along with the fall in its natural production led to a growing interest in cultivation techniques (Bach et al., 2021) and encouraged truffle growers and researchers to deeper investigate factors that could affect and improve *T. magnatum* productivity. In this context, microbial communities deserve particular attention, since they are of undoubted importance for the ecology and life cycle of the fungus. Interestingly, a potential link between microbial taxa and truffle ground productivity was observed and specific bacteria (e.g., *Moraxella osloensis*) have been identified as promising *T. magnatum* “productivity markers” (Mello et al., 2010; Sillo et al., 2022). However, to date, studies on this subject are still very few and several aspects related to the role of bacteria in truffle biology should be properly investigated (Splivallo et al., 2019; Monaco et al., 2021b). For example, since the truffle mycelium can influence the composition and activity of soil microbial communities (Napoli et al., 2010; Mello et al., 2013; Marozzi et al., 2022), it should be clarified whether the taxa retrieved in “productive soils” actually contribute to a higher truffle production or, on the contrary, their occurrence is a consequence of *T. magnatum* presence. Another important open question concerns the nitrogen-fixing activity and the resulting involvement of bacteria in truffle nutrition, development, and maturation. Indeed, as previously described, potential nitrogen-fixing bacteria (mainly represented by *Bradyrhizobium* and *Rhizobium* genera) usually occur in *Tuber* ascomata, regardless of their maturity level (Barbieri et al., 2007). The bacterial ability to modify nutrient availability in the soil could be of fundamental importance for the fungus, especially during the early stages of ascocarp formation (Barbieri et al., 2007; Vahdatzadeh et al., 2015; Monaco et al., 2020a). In 2010, Barbieri and colleagues (Barbieri et al., 2010) evaluated the nitrogenase activity and demonstrated for the first time the expression of *nifH* genes from *Bradyrhizobia* in *T. magnatum* ascomata. Nevertheless, to date there is no coherent direct evidence to support that bacterial nitrogen fixation occurs during truffle development and it remains to be proved that nitrogen fixed by bacteria within truffle ascocarps is subsequently really transferred to the fungus (Vahdatzadeh et al., 2015; Marozzi et al., 2022).

Therefore, a more detailed understanding of the relationship between truffles and their environment is required. In order to define a possible link between productive seasons and soil microbial composition, it could be interesting to characterise the microbial communities within truffle sites over time. The identification of microorganisms actually able to promote *T. magnatum* formation may represent an important advance for the development and/or improvement of the white truffle cultivation, with significant repercussions in the field of truffle farming (Perlińska-Lenart et al., 2020; Monaco et al., 2020a; Marozzi et al., 2022). Besides being potential “productivity markers,” bacteria appear to be also a promising tool for tracing the geographic origin of truffles (Monaco et al., 2021b; Niimi et al., 2021a). Indeed, since the (economic) value of the prized *T. magnatum* can vary in relation to fruiting body provenience (Sillo et al., 2022), an increasing interest to define a reliable tracking system is emerging. Researchers have so far mainly focused on other aspects, including the analysis of intraspecific genetic variability (Mello et al., 2005; Monaco et al., 2021b), population genetic structure (Rubini et al., 2005; Belfiori et al., 2020), antioxidant compounds (Vita et al., 2018), transcriptomic, proteomic, and volatilomic profiles (Vita et al., 2020), as well as morphological traits, such as peridium thickness (Monaco et al., 2021a). The cost of truffle is directly influenced also by fruiting body aroma, a unique mixture of volatile organic compounds (VOCs) partially synthesised by bacteria. Interestingly, some of these chemicals are common to several *Tuber* species and might be of mixed truffle and microbial origin, whereas others are species-specific and could derive only from microbes (Vahdatzadeh et al., 2019). In fact, it is known that besides a core microbiota shared between diverse *Tuber* species, “non-fixed” bacterial taxa complete the composition of the truffle-associated microbial communities and contribute to determine the observed inter- and intra-specific differences (Monaco et al., 2021b). Hence, it is reasonable to search for potential markers of *T. magnatum* geographic origin among these variable bacteria. Of course, to be a good biomarker, a microbial taxon should have specific features. Firstly, it should be almost constantly present in truffles coming from a particular geographical area and absent in fruit bodies with a diverse provenience or, at least, show significantly different abundance values. A “tracer microorganism” also needs to be well represented within the examined microbial community so that it can be easily detected through the available analysis methods. In addition, the taxonomic level of investigation should be as specific as possible. Niimi et al. (2021a) identified an OTU belonging to the genus *Pseudomonas* as a potential marker of geographic origin of some Hungarian white truffles. Despite this encouraging result, there are still some difficulties and limitations to take into account: (1) the reduced number of studies and analysed samples that do not allow appropriate comparisons and (2) the small-scale heterogeneity in truffle microbiota composition (Monaco et al., 2021b). Moreover, since environmental factors, such as temperature, humidity, soil properties, microclimatic conditions, and snow cover (Monaco et al., 2020b), significantly affect the structure of microbial communities, even little variations can determine important changes in the truffle microbiota.

In conclusion, although further studies on the role of bacteria, microbiota composition and factors driving the establishment of a specific bacterial signature in *T. magnatum* are required, the analysis of microbial taxa appears to be a promising tool for the identification of *Tuber* productivity markers and geographic origin indicators, with a considerable impact on truffle farming, industry, and trade.

## Author contributions

AB, AM, and PM: conceptualization. PM: writing–original draft preparation and visualisation. AB, AM, GN, and PM: writing–review and editing. AB and AM: supervision. All authors contributed to the article and approved the submitted version.

## Conflict of interest

The authors declare that the research was conducted in the absence of any commercial or financial relationships that could be construed as a potential conflict of interest.

## Publisher’s note

All claims expressed in this article are solely those of the authors and do not necessarily represent those of their affiliated organizations, or those of the publisher, the editors and the reviewers. Any product that may be evaluated in this article, or claim that may be made by its manufacturer, is not guaranteed or endorsed by the publisher.

## Footnotes

^1^ https://www.unesco.it/it/PatrimonioImmateriale/Detail/1370
